# Decoding and overcoming T cell exhaustion: Epigenetic and transcriptional dynamics in CAR-T cells against solid tumors

**DOI:** 10.1016/j.ymthe.2024.04.004

**Published:** 2024-04-06

**Authors:** Taeyoung Ahn, Eun-Ah Bae, Hyungseok Seo

**Affiliations:** 1 Laboratory of Cell & Gene Therapy, Institute of Pharmaceutical Sciences, Seoul National University, Seoul 08826, Republic of Korea; 2 Laboratory of Immunology, Department of Molecular Medicine and Biopharmaceutical Sciences, Graduate School of Convergence Science and Technology, and College of Pharmacy, Seoul National University, Seoul 08826, Republic of Korea

**Keywords:** CAR-T, T cell exhaustion, transcription factor, epigenetic regulation, genomic engineering

## Abstract

T cell exhaustion, which is observed in various chronic infections and malignancies, is characterized by elevated expression of multiple inhibitory receptors, impaired effector functions, decreased proliferation, and reduced cytokine production. Notably, while adoptive T cell therapies, such as chimeric antigen receptor (CAR)-T therapy, have shown promise in treating cancer and other diseases, the efficacy of these therapies is often compromised by T cell exhaustion. It is imperative, therefore, to understand the mechanisms underlying this exhaustion to promote advances in T cell-related therapies. Here, we divided exhausted T cells into three distinct subsets according to their developmental and functional profiles: stem-like progenitor cells, intermediately exhausted cells, and terminally exhausted cells. These subsets are carefully regulated by synergistic mechanisms that involve transcriptional and epigenetic modulators. Key transcription factors, such as TCF1, BACH2, and TOX, are crucial for defining and sustaining exhaustion phenotypes. Concurrently, epigenetic regulators, such as TET2 and DNMT3A, shape the chromatin dynamics that direct T cell fate. The interplay of these molecular drivers has recently been highlighted in CAR-T research, revealing promising therapeutic directions. Thus, a profound understanding of exhausted T cell hierarchies and their molecular complexities may reveal innovative and improved tumor treatment strategies.

## Graphical abstract

**Figure:**
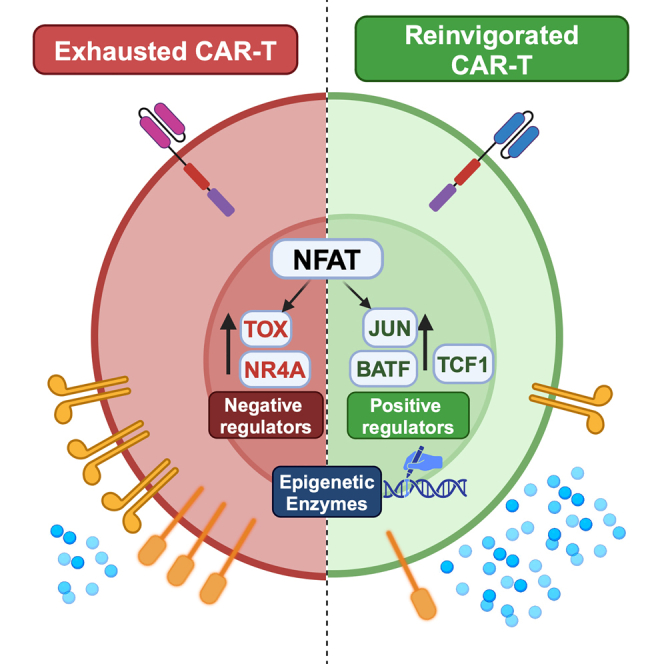

## Teaser

Seo and colleagues dissect T cell exhaustion and categorize exhausted T cells into three subsets, revealing the roles of key transcription and epigenetic modulators in CAR-T therapy effectiveness. This study unveils pathways for enhancing T cell therapies, offering new avenues for cancer treatment advancements.

## Introduction

T cell exhaustion was originally described in mouse models of chronic viral infection; in these models, the persistent stimulation of T cell receptors (TCRs) by viral antigens was identified as a key driver of T cell exhaustion.^1^^,^^2^^,^^3^ This same mechanism of constant TCR engagement was later observed in tumor environments, leading to the conclusion that T cell exhaustion also occurs in mouse and human cancer (Table 1).^4^^,^^5^ While chronic stimulation is the fundamental cause of T cell exhaustion in both contexts, the specific pathways that lead to exhaustion differ due to external factors, such as metabolic disparities and unique aspects of the tumor microenvironment.^6^^,^^7^^,^^8^ Despite these differences, the resulting decrease in T cell function is a common hallmark of T cell exhaustion, reflecting a conserved state of T cell impairment in both chronic infections and cancer.^8^^,^^9^ Understanding T cell exhaustion has significantly contributed to the development of treatments aimed at mitigating or reversing T cell dysfunction, which thereby enhances the efficacy of cancer immunotherapies. These findings highlight the translational value of the comprehensive characterization of T cell responses in ongoing efforts to treat cancer.

**Table 1:** Comparative overview of CD8+ T cell exhaustion in chronic viral infections and cancer microenvironments

Parameters	T cell exhaustion in	
	Chronic viral infections	Cancers (tumor microenvironment)
Antigen persistence	continual presence of viral antigens	varied; can include tumor-specific antigens and neoantigens
Inhibitory receptors	high expression (e.g., PD-1, CTLA-4)	high expression, potentially with different patterns
Immune environments	dominated by viral evasion tactics; often systemic	complex with cellular components like regulatory T cells (Tregs), myeloid-derived suppressor cells (MDSCs)
Antigen diversity	complex with cellular components like Tregs and MDSCs	high due to tumor heterogeneity
Effector functions	reduced due to chronic stimulation	reduced due to chronic stimulation
Metabolic state	can be altered by long-term infection	hypoxia and nutrient competition
Therapeutic implications	focus on restoring function; antiviral therapies	immune checkpoint blockade, vaccines, targeted therapies

This table outlines the mechanisms of T cell exhaustion in chronic viral infections versus cancers within the tumor microenvironment. It highlights the differences in antigen persistence, expression of inhibitory receptors, and immune environments between these two settings. Chronic viral infections feature continual viral antigen presence and high inhibitory receptor expression in a systemically altered immune environment. In contrast, cancers present a mix of tumor-specific antigens and a complex microenvironment with various suppressive cells and factors alongside high, potentially diverse, inhibitory receptor expression.

Recent advances in understanding T cell biology have shown that both chimeric antigen receptor (CAR)-T cells and CD8+ T cells can recognize specific antigens, as well as antigens that are presented by major histocompatibility complex class I molecules, through their immunoreceptors (CAR and TCR, respectively).^10^^,^^11^^,^^12^^,^^13^^,^^14^ Signaling cascades are then initiated by an intracellular immunoreceptor tyrosine-based activation motif in CD3 or the TCR zeta chain. These cascades involve proximal signaling kinases, notably ZAP-70 and LCK, and ultimately lead to the activation of PLCγ. As a result, an elevation in intracellular calcium concentrations occurs. Driven by calcineurin, cytosolic nuclear factor of activated T cells (NFAT) then translocates to the nucleus, where it becomes activated.^15^^,^^16^^,^^17^

**Figure 1:**
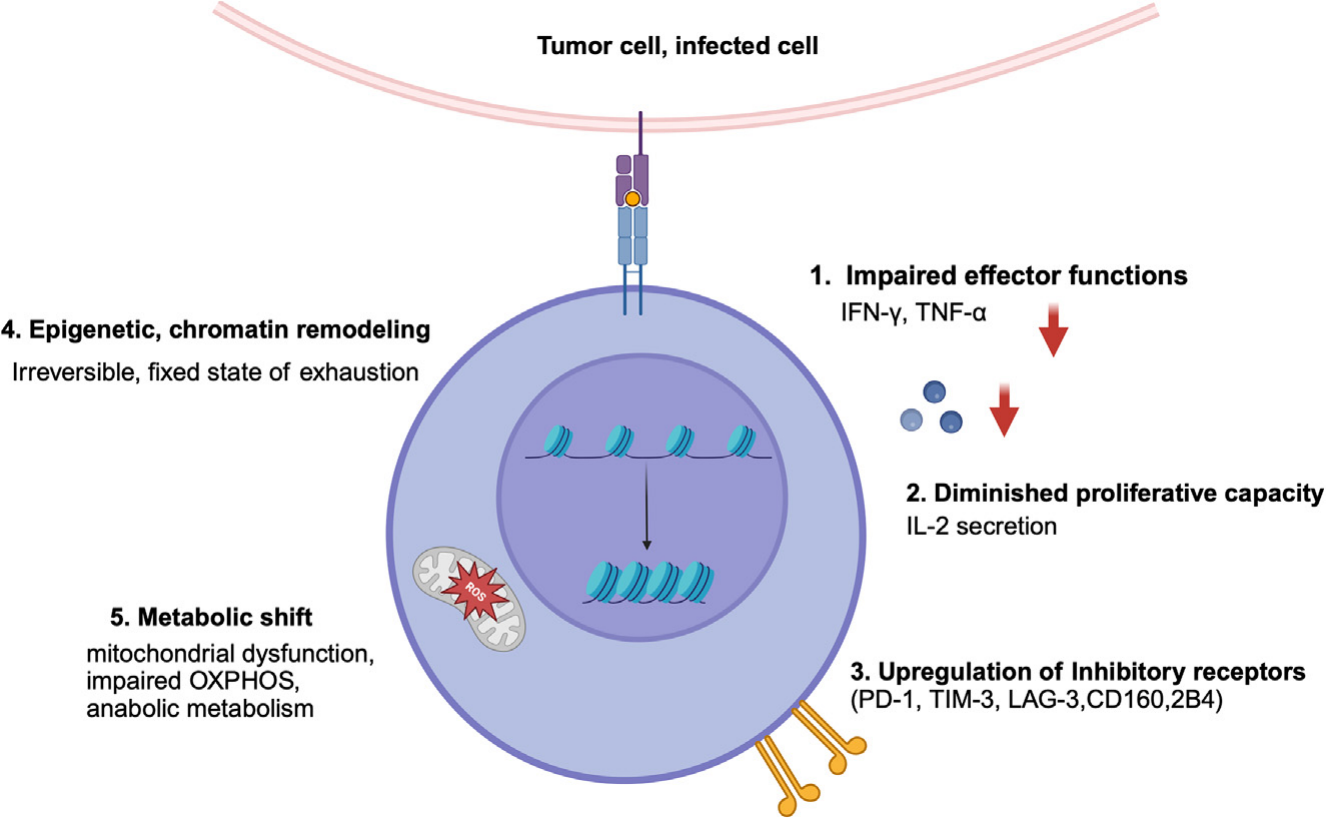
Overview of distinct properties of exhausted (dysfunctional) CD8+ T cells This figure provides a comprehensive overview of the key attributes and stages of CD8+ T cell exhaustion in response to prolonged antigen exposure. It illustrates the progression from an initial state of impaired effector functions, characterized by reduced secretion of cytokines such as IFN-γ and TNF-α, to a decline in proliferative capacity and IL-2 production. The figure also highlights the upregulation of inhibitory receptors (e.g., PD-1, TIM-3, LAG-3) and the deep epigenetic and chromatin remodeling that cements the T cell in an exhausted state. Lastly, it depicts the metabolic shift toward mitochondrial dysfunction and altered metabolism, underpinning the cells’ diminished response to therapeutic interventions.

Concurrently, NFAT interacts with activator protein 1 (AP-1), which is a heterodimeric transcription factor that is composed of proteins such as c-JUN and c-Fos and members of the bZIP family of transcription factors. This interaction drives T cell activation and differentiation in a context-specific manner.^18^^,^^19^ Notably, proteins that constitute the AP-1 transcription factor can be modulated by signals that are initiated by various costimulatory molecules and receptors, including CD28, integrins such as LFA-1, and coreceptors such as CD4.^20^^,^^21^ Specifically, when the TCR is engaged in the absence of appropriate costimulatory signaling, NFAT activation occurs without concurrent AP-1 activation. This subsequently results in T cell anergy.^22^^,^^23^ After activation, even as T cells differentiate into effector T cells, progressive attenuation of T cell function is observed under conditions where antigens persist and continuously stimulate T cells via cognate receptors. This intrinsic impairment of T cell function is termed T cell exhaustion, hypo-responsiveness, or dysfunction. Notably, such phenomena have been documented in both tumor models and chronic virus infection models.^6^^,^^24^ T cell exhaustion is characterized by heightened expression of inhibitory receptors, reduced effector function and proliferative capacity, and attenuated activation signaling and metabolic alterations^7^ (Figure 1). The transcriptional and epigenetic profiles of exhausted T cells highlight that T cell exhaustion represents a distinct cellular state that is distinct from the cellular state of effector, memory, or anergic CD8+ T cells.^9^^,^^25^ This transition toward exhaustion progresses gradually and predominantly relies on TCR signaling. While initially reversible, over time, this state becomes irreversible.^26^ Intriguingly, phenomena reminiscent of exhaustion have been observed in both a mouse model of chronic LCMV infection and various murine tumor models. With the successful progression of CAR-T cell clinical trials, recent studies have revealed signs of human T cell exhaustion in the context of CAR-T cells. Notably, CAR-T-cell exhaustion was observed after continuous antigen exposure in the CAR stress test, which was designed to evaluate the ability of cells to manage suboptimal tumor doses. Interestingly, this exhaustion can also be triggered without antigen exposure by antigen-independent intrinsic tonic signaling through the CAR.^27^^,^^28^

In this manuscript, we elucidate the mechanisms by which CD8+ T cells transition to a dysfunctional state, termed “exhausted CD8+ T cells,” by focusing on transcriptional and epigenetic parameters and performing various mouse and human studies. Based on these findings, we also summarize recent studies on exhausted T cells in the context of CAR-T cell development and propose future directions for the development of next-generation adoptive T cell therapies.

## Subsets of exhausted T cells

**Figure 2:**
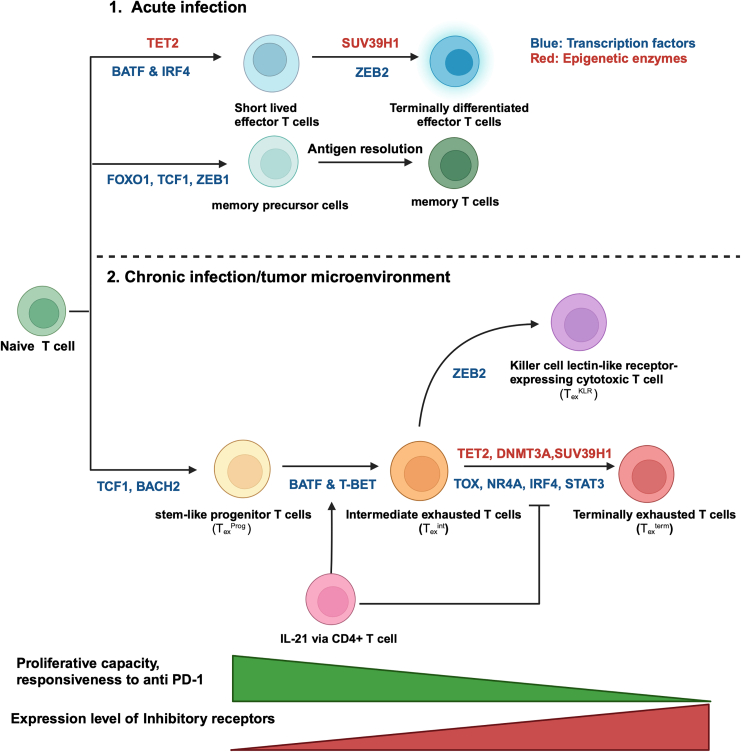
Differentiation trajectories and subsets of CD8+ T cells exhibiting exhaustion in persistent antigenic environments This figure delineates the progression of CD8+ T cell exhaustion during sustained antigen exposure, typically observed in chronic infections. It specifies the unique surface proteins and transcription factors characterizing each T cell subset. In the initial phase of chronic infection, effector-like T cells, which bear resemblance but are distinct from their counterparts in acute infections, give rise to T_ex_^Prog^ cells. These cells possess the capacity for self-renewal and eventually evolve into two distinct progeny subsets, T_ex_^int^ and T_ex_^term^ cells, in response to continuous antigen presence. Notably, the recent discovery of T_ex_^KLR^, an alternative terminal subset, points to a non-linear differentiation pathway in T cell exhaustion, as represented by the dotted trajectory.

The differentiation of T cells from a naive state to effector and memory states is well established, and each state is characterized by distinct markers and functions.^29^^,^^30^^,^^31^ In the context of chronic infection and cancer, this classical pathway is mirrored and divergent in subsets of exhausted T cells, which exhibit altered characteristics and functions due to persistent antigen stimulation (Table 2).^2^^,^^6^^,^^25^^,^^32^ Numerous studies have investigated the subpopulations and differentiation pathways that are associated with T cell exhaustion, and these subpopulations can be broadly classified into three primary subsets: (1) stem-like progenitor exhausted T (T_ex_^Prog^) cells, (2) intermediate exhausted T (T_ex_^int^) cells, and (3) terminally exhausted T (T_ex_^term^) cells (Figure 2; Table 2).^8^^,^^33^^,^^34^^,^^35^^,^^36^^,^^37^^,^^38^^,^^39^ In the following paragraph, we provide an overview of recent findings that are related to the key characteristics of exhausted T cell subsets in the context of chronic infection and tumors.^33^

**Table 2:** Distinguishing features of exhausted T cell subsets and their normal counterparts

Subset	Defining markers	Normal counterpart	Counterpart markers	Functionality difference
Stem-like progenitor T (T_ex_^Prog^) cells	PD-1 high, TCF1 +, CXCR5+, etc.	naive and stem-like memory T cells	TCF-1+, CD62L+, CCR7+, etc.	T_ex_^Prog^ cells have impaired response to chronic antigen stimulation and maintain a state of exhaustion, while normal counterparts have full functionality and respond to acute antigen stimulation
Intermediate exhausted T (T_ex_^int^) cells	PD-1 high, TIM-3+, etc.	effector memory T cells	CD44 high, CD62L low (in mice), etc.	T_ex_^int^ cells show signs of exhaustion with diminished effector functions compared to fully functional effector memory T cells
Terminally exhausted T (T_ex_^term^) cells	PD-1 high, LAG-3+, CD160+, CD69+, etc.	terminally differentiated effector T cells	KLRG1+, CD57+, etc.	T_ex_^term^ cells have severely limited proliferative and effector capabilities, unlike their effector T cell counterparts that can effectively clear infections or tumor cells

This table classifies subsets of exhausted T cells, defined by their expression markers, comparing them with their normal counterparts and discussing functionality differences. It distinguishes between stem-like exhausted T (T_ex_^Prog^) cells with high PD-1 and other markers indicating a stem cell-like state and intermediate exhausted T (T_ex_^Int^) cells with high PD-1 and TIM-3 expression. For each subset, the table identifies normal counterparts based on marker expression and outlines differences in functionality, emphasizing the impaired response of Tex^Prog^ cells to chronic stimulation and the diminished efficacy of T_ex_^Int^ cells.

First, T_ex_^Prog^ cells**,** which are characterized by the TCF1+PD-1+CD8+ phenotype, are known for their self-renewal and proliferative capacities.^36^ Due to its inherent qualities, this cell subset is crucial for maintaining long-term immune responses to antigens.^35^^,^^38^^,^^40^ This progenitor subset is generated in a TCR-signaling-dependent manner. Notably, it can develop rapidly, emerging within days during tumorigenesis.^26^^,^^41^ Recent studies have revealed that impaired effector function and changes in dysfunction-associated epigenetic programming occur even before cell division.^42^ T_ex_^Prog^ cells have the potential to differentiate into T_ex_^int^ cells and T_ex_^term^ cells. The depletion of T_ex_^Prog^ cells has been associated with decreased tumor control, reduced generation of T-bet-expressing transitory effector-like T cells, and inhibited CD8+ T cell expansion. This subset has been shown to be a trigger for bursts of T cell proliferation in response to anti-PD-1 immune checkpoint blockade (ICB) in both LCMV and tumor mouse models.^32^^,^^36^^,^^42^^,^^43^^,^^44^^,^^45^ T_ex_^Prog^ cells are thought to either reside in lymphoid organs or circulate, as observed in the LCMV Cl13 model.^34^^,^^44^ When exposed to antigens, these cells proliferate and progressively irreversibly differentiate into T_ex_^int^ cells and T_ex_^term^ cells over time.

In mouse tumor microenvironments, similar to those in the LCMV Cl13 model, CD8+ T cells in the early phase (day 8) exhibit reversible impairment, whereas CD8+ T cells in the late phase (day 35) exhibit irreversible dysfunction from which they cannot be rescued.^26^ Notably, the epigenetic reprogramming of T_ex_^Prog^ cells occurs quickly.^41^^,^^42^ It has been reported that these cells are unable to generate effector T cells and are primarily implicated in producing dysfunctional or exhausted lineages instead.^46^

T_ex_^int^ cells, which are also known as the proliferating transitory T cell subset, are offspring that differentiate from T_ex_^Prog^ cells.^34^ These cells exhibit a unique phenotype that is characterized by the expression of CX3CR1 and PD-1; this phenotype is observed in chronic virus infection mouse models.^33^ Moreover, comparable functional T_ex_^int^ cells have been identified among human tumor-infiltrating CD8+ T cells.^47^ The progression from T_ex_^Prog^ cells to T_ex_^int^ cells seems to rely on CD4+ T cell-mediated interleukin (IL)-21 signaling.^48^ Compared to T_ex_^term^ cells, this proliferating T_ex_^int^ cell subset displays enhanced expression of effector cytokines (interferons [IFNs], tumor necrosis factors [TNFs], and IL-2) and cytotoxic molecules (perforin and granzymes). This subset proliferates in response to ICB and facilitates enhanced viral control.^33^^,^^48^ T_ex_^int^ cells, while unable to revert to T_ex_^Prog^ cells, have the potential to develop into T_ex_^term^ cells. These cells can be distinguished from the short-lived effector T cells that are typically observed during acute infections, both epigenetically and transcriptionally, as evidenced in previous studies.^33^^,^^48^^,^^49^ This subset, while incapable of reverting to T_ex_^Prog^ cells, demonstrates progression toward T_ex_^term^ cells, which are characterized by CD101+ expression and a terminal exhaustion phenotype. The unique transitional role of T_ex_^Prog^ cells compared with that of T_ex_^term^ cells, coupled with their distinct epigenetic and transcriptional profiles, highlights their critical function in the immune response during chronic infections.

T_ex_^term^ cells are characterized by their severely decreased proliferation and functional capacities. Chronic antigen exposure not only compromises CD8+ T cell function but also leads to irreversible epigenetic reprogramming, resulting in deeply embedded dysfunction. These cells are characterized by a distinct profile of surface proteins, such as CD101, TIM-3, LAG-3, CD160, and CD69, as well as elevated levels of transcription factors, such as TOX, NR4A, PRDM1, and EOMES. T_ex_^term^ cells exhibit resistance to ICB therapy, suggesting that preventing their progression to a permanently dysfunctional state could enhance the therapeutic potential of CAR-T cells.^34^^,^^50^^,^^51^^,^^52^ This strategy is supported by ongoing research, which suggests new directions for improving CAR-T cell efficacy in cancer treatment. While a linear differentiation trajectory mediated by TCR engagement was once a widely accepted concept, emerging findings are challenging this perspective. A recently identified subset of exhausted T cells in the context of LCMV chronic infection, termed T_ex_^KLR^ cells, which express the killer cell lectin-like receptor (KLR) along with natural killer (NK)-associated genes such as NKG2A and CD94, diverges from the previously understood trajectory. Instead of expressing markers such as NR4A2, CD160, LAG-3, and CD244, which are upregulated in T_ex_^term^ cells, these T_ex_^KLR^ cells seem to originate from T_ex_^int^ cells alongside T_ex_^term^ cells.^49^^,^^53^ As such, a range of models, including linear, linear-non-progression, and bifurcation trajectories, are being proposed to explain these dynamics.^48^^,^^49^^,^^53^^,^^54^ Notably, exhausted CAR-T cells expressing NK-associated genes, similar to T_ex_^KLR^ cells, were observed in patients with cancer, highlighting the need for a deeper understanding of CAR-T cell exhaustion and dysfunction trajectories.^55^ However, the exact mechanism underlying the exhaustion of CAR-T cells and tumor-infiltrating T cells requires further elucidation.

## Roles of transcription factors in T cell exhaustion

TCF1, which has been identified as a hallmark transcription factor in T_ex_^Prog^ cells, becomes activated when tumor-specific CD8+ T cells are stimulated by NFAT and BATF.^35^^,^^41^ The subsequent activation of BACH2 and TCF1 results in T_ex_^Prog^ cell differentiation.^56^^,^^57^ TCF1 is predominantly expressed in memory precursor cells during the resolution of acute immune responses, and it is known to contribute to the maintenance of T cell quiescence.^37^^,^^58^ The many roles and functions of TCF1 were extensively described in another review.^59^ While genetic knockout (KO) of TCF1 or selective depletion of TCF1+ T cells does not inhibit normal T cell expansion during early phases of infection, the lack of TCF1 significantly decreases T cell persistence and viral control during chronic viral infection.^35^^,^^38^ In mouse tumors, deletion of TCF1+PD-1+ tumor-infiltrating lymphocytes suppressed antitumor responses after the administration of immunotherapy.^35^^,^^36^^,^^38^ As T cell exhaustion progresses, the expression of TCF1 decreases, especially after T cell proliferation and subsequent irreversible epigenetic reprogramming via methylation.^34^

BACH2 is a pivotal transcription factor that is crucial for memory T cell differentiation during acute infection, and it performs this function by moderating TCR signaling and regulating the accessibility of IRF4 and AP-1 to enhancers.^41^ Recent observations indicate that, in tandem with ID3 and TCF1, BACH2 expression is enriched in T_ex_^Prog^ cells during chronic infection, but it is not expressed in T_ex_^term^ cells.^57^ Intriguingly, overexpression of BACH2 leads to the upregulation of genes that are characteristic of stem-like T cells, such as TCF1, ID3, and LY108, and simultaneously suppresses the expression of inhibitory receptors, such as TIM-3 and PD-1, and the transcription factor TOX.^57^ In contrast, BACH2 KO exerts a minimal effect on the phenotype of T_ex_^term^ cells. However, it compromises the maintenance and differentiation of T_ex_^Prog^ cells; these results highlight the essential role of BACH2 in maintaining prolonged immune responses by promoting T_ex_^Prog^ cell function and inhibiting terminal differentiation.^57^^,^^60^

MYB has been recognized as a crucial factor that determines the differentiation and maintenance of T_ex_^Prog^ cells. It not only promotes the transcription of TCF1 but also simultaneously inhibits ZEB2, which is a transcription factor that is known to drive further differentiation.^61^^,^^62^ Given these multifaceted functions, MYB serves as a regulatory checkpoint preserving the differentiation of T_ex_^Prog^ cells.^39^^,^^61^ Interestingly, MYB expression is regulated in a dose-dependent manner by TCR signaling, and it is prominently expressed in quiescent CD62L+ T_ex_^Prog^ cells that are located in the spleen and lymph nodes during chronic infection.^62^ This subset of MYB-expressing cells is especially susceptible to immune checkpoint inhibitors, highlighting the pivotal role of MYB in sustaining the pool of ICB-responsive T_ex_^Prog^ cells. However, it is noteworthy that the expression of MYB in this subset was not very consistent with the previously described expression of Ly108 and CD69 in T_ex_^Prog^ cells.^34^^,^^62^ This inconsistency could occur because of different marker proteins were used in different studies to categorize T cell subsets during chronic infection. Thus, additional investigations are necessary to delineate the subpopulations that are related to lymph-node-resident MYB-expressing CD62L+ T_ex_^Prog^ cells.

T-BET and EOMES have emerged as pivotal transcription factors that enhance the effector functions of T_ex_^int^ cells. As T_ex_^Prog^ cells evolve into T_ex_^int^ cells, the expression of T-bet increases. T-bet aids in maintaining virus-specific responses during chronic virus infection and suppresses inhibitory receptors such as PD-1.^63^ In chronic infection models, a complex interaction between T-BET and EOMES is evident. Notably, T_ex_^Prog^ cells exhibit reduced T-bet expression while maintaining increased EOMES levels of expression. As differentiation toward T_ex_^int^ cells progresses, an increased T-BET/EOMES expression ratio is observed. When T_ex_^int^ cells undergo terminal differentiation, EOMES levels are restored, while T-bet expression is decreased.^34^ The downregulation of T-bet triggers swift differentiation toward T_ex_^term^ cells in early stages and results in a substantial decrease in total effector CD8+ T cells, highlighting the crucial role of T-bet in guiding the development, survival, and function of the T_ex_^int^ cell subset.^64^

NFAT, IRF4, and BATF constitute a crucial axis that is related to T cell exhaustion. In the context of chronic infections and tumors, persistent antigen stimulation induces elevated expression of TCR-responsive elements such as NFAT and IRF4. When IRF4 is suppressed in CD8+ T cells during chronic infection, there is a corresponding reduction in the expression of inhibitory receptors, such as 2B4, TIM3, TIGIT, LAG3, and PD-1. Concurrently, compared with their wild-type counterparts, these cells exhibit increased secretion of IFN-γ and TNF-α and a metabolic shift toward enhanced anabolic metabolism and oxidative phosphorylation. The synergistic expression and interaction of IRF4 with BATF, which is driven by a positive feedback loop with NFAT, promotes the differentiation of T_ex_^term^ cells in the context of persistent infection.^65^ Conversely, it is crucial to recognize the significant roles that BATF and IRF4 play in differentiating effector T cells and T_ex_^int^ cells during chronic infection. While IRF4 is not critical for the initial proliferation or expression of effector molecules during acute infection, it is indispensable for long-term clonal expansion and the maintenance of effector function in a TCR affinity-dependent manner.^66^ During chronic infection, IL-21, which is crucial for the transition to of cells to the cytolytic T_ex_^int^ phenotype, triggers the expression of BATF.^67^ BATF synergizes with IRF4, which is induced in manner that depends on TCR signaling, and binds to the AICE site.^68^ The synergistic interaction promotes the transition to of T cells to the cytolytic T_ex_^int^ phenotype, highlighting their combined effect on modulating T cell effector function.^67^ Importantly, in CAR-T cell research, overexpression of BATF has been associated with decreased expression of TOX, PD-1, and other inhibitory receptors, and when BATF is paired with IRF4, this modification preserves long-term effector functions, inducing marked antitumor effects.^50^

The TOX and NR4A families have been shown to be pivotal transcription factors that regulate T cell exhaustion. While they are non-essential for the differentiation and development of effector and memory T cells during acute infection, they play crucial roles in the context of chronic infection and tumors. In such environments, these factors are upregulated in exhausted T cells and tumor-specific T cells.^50^^,^^69^^,^^70^^,^^71^^,^^72^^,^^73^ Early stages of chronic infection trigger the induction of TOX expression through TCR-signaling-induced Ca^2+^ flux, leading to the binding of NFAT2 (*Nfatc1*) at the *Tox* locus. Once T cells become irreversibly exhausted, TOX expression persists in a calcineurin-independent manner.^74^ Downregulation of TOX results in decreased expression of inhibitory receptors (TIM-3, LAG-3, and PD-1) and augmented effector functions and antitumor effects. However, ablation of TOX severely inhibits the persistence of CD8+ T cells during chronic infection and under tumor conditions.^71^^,^^73^^,^^74^ It is theorized that TOX-mediated exhaustion prevents T cell overstimulation and activation-induced cell death due to chronic antigen stimulation, promoting long-term T cell persistence. TOX has been conclusively associated with the development of TCF1+ T_ex_^Prog^ cells and increased expression of inhibitory receptors such as PD-1 and LAG-3 during terminal exhaustion. TOX modifies transcription and chromatin accessibility, leading to irreversible T cell exhaustion.

In parallel with TOX, NR4A upregulation is ubiquitous among various exhausted T cell populations in the context of chronic viral infection, tumors, and CAR-T cells.^51^ Notably, without its partner transcription factor AP-1, NFAT alone induces the expression of NR4A, which is another vital transcription factor that is involved in terminal T cell exhaustion. NR4A and TOX form a symbiotic positive feedback loop that regulates the expression of inhibitory receptors and modifies the chromatin accessibility of transcription factors that are essential for promoting effector functions, such as bZIP and nuclear factor κB (NF-κB). Notably, CAR-T cells with triple KO of NR4A family transcription factors (Nr4a1, Nr4a2, and Nr4a3) exert enhanced antitumor effects, increased expression of effector cytokines (IFNs, TNFs, and IL-2) and cytotoxic molecules (perforin and granzymes), decreased expression of inhibitory receptors, and augmented chromatin accessibility at motifs that are related to transcription factors that are related to effector function, such as AP-1 and NF-κB, in a mouse tumor model.^51^^,^^71^

STAT3 has been recently shown to play crucial roles in T_ex_^term^ cell development in cancer.^75^ Various cytokines are involved in STAT3 activation during CD8+ T cell activation and exhaustion^67^^,^^76^*.* IL-10 and IL-21, but not IL-6, activate STAT3, promote tumor-specific T_ex_^term^ cell-associated gene expression, and suppress T_ex_^Prog^ cell-related gene expression, resulting in the development and enhanced effector functions of T_ex_^int^ cells in tumors.^75^ The IL-21-STAT3-BATF pathway is necessary for sustaining the effector function of CD8+ T cells during chronic infection.^67^ Building on this understanding, recent research has provided deeper insights into the function of STAT3.^75^ In the tumor microenvironment, STAT3, which is predominantly activated by IL-10 and IL-21, but not by IL-6, enhances the effector function and survival of T_ex_^term^ cells, thereby improving tumor regulation.^67^^,^^75^ This study also highlights the transcriptional regulatory role of STAT3 in promoting effector-function-related gene expression and suppressing T_ex_^Prog^ gene expression; thus, STAT3 cooperates with BATF and IRF4 to activate chromatin.^75^ These findings emphasize the critical role of STAT3 in T_ex_^term^ cell development in cancer, offering valuable perspectives for improving immunotherapies.

## Epigenetic reprogramming of CD8+ T cells in chronic infection and tumors

TET2 is a methylcytosine dioxygenase that functions as an epigenetic modulator by facilitating DNA demethylation and converting 5-methylcytosine to 5-hydroxymethylcytosine during DNA replication.^77^^,^^78^^,^^79^ TET2 plays an integral role in effector T cell differentiation through demethylation, and disruption of TET2 has been correlated with an increased proportion of CD127+ and KLRG1− T memory precursor cells.^80^^,^^81^ Notably, TET2-KO CAR-T cells have demonstrated marked therapeutic effects in clinical patients, achieving complete remission and exhibiting enhanced antitumor effects.^81^^,^^82^ Moreover, TET2 ablation in CAR-T cells alters the epigenetic state of the T cells, leading to an increase in proportion of cells with the central memory-like phenotype [CCR7+CD45RO+].^80^^,^^81^ These TET2-KO CAR-T cells retain high proliferative properties over time, and interestingly, while TET2 ablation does not affect the expression of inhibitory receptors, an increase in the expression of cytolytic proteins, such as CD107a, granzyme B, and perforin, has been observed, despite a decrease in effector cytokines.^81^^,^^82^ Nevertheless, these cells continue to exhibit potent antitumor activity, which was substantiated by both *in vivo* studies and clinical applications.^81^

Considering several aspects of the role of TET2 in T cell differentiation and the feasibility of using TET2 as a gene-editing target for adoptive cell therapy are crucial. Distinct from canonical CAR-T cells, which become dysfunctional upon persistent antigen exposure, TET2-KO CAR-T cells exhibit hyperproliferative clonal expansion in an antigen-independent manner dependent on the 4-1BB CAR costimulatory domain.^82^ However, the findings related to knocking out TET2 in CAR-T cells are not consistent. While some studies suggest an increase in central memory-like CAR-T cells, others have observed an enrichment of gene sets related to lymphoma and leukemia rather than memory-associated gene sets.^80^^,^^81^^,^^82^ Moreover, it has been observed that the KO of TET2 in CAR-T cells does not impact the expression of TCF1, which is a marker that is found in memory precursor T cells and stem-like T cells. An increased incidence of somatic mutations in TET2-KO CAR-T cells also indicates a compromise in gene integrity. The properties of TET2-KO CAR-T cells are functionally and phenotypically divergent from those of memory, effector, and exhausted T cells. The pivotal role of TET2 in the transition from naive to effector differentiation, along with its effect on central memory T cell markers such as CCR7 and CD127, is noteworthy. Given this context, it is imperative to thoroughly investigate whether the transcriptional and epigenetic profiles of TET2-KO CAR-T cells are in true alignment with those of canonical memory T cells.^83^ In this context, the transcription factor BATF3 emerges as a significant element in the landscape of T cell biology, and discussion of this factor here is warranted. BATF3 is recognized for its role in promoting the proliferation and differentiation of memory T cells, with substantial implications for T cell exhaustion. The effect of BATF3 is particularly pronounced in the context of TET2-KO CAR-T cells, suggesting that BATF3 plays a multifaceted role in both memory formation and exhaustion.^82^ This finding highlights the potential of epigenetic modulation strategies in CD8+ T cells, aiming to increase antitumor responses and the success of adoptive cell therapy initiatives.

DNMT3A functions as a *de novo* DNA methyltransferase, regulating epigenetic modulation during the effector-to-exhaustion transition that is observed in LCMV chronic infection.^52^ Notably, exhaustion-associated epigenetic reprogramming, which is mediated by DNMT3A, occurs in response to sustained TCR engagement. This finding differs from the DNA methylation profiles of memory CD8 T cells that are observed during acute infection. A conditional deletion of DNMT3A in CD8+ T cells promotes prolonged maintenance of effector functions as well as markedly decreases epigenetic silencing at various loci, including *Ifnγ*, *Tcf7*, *Ccr7*, and *Tbx21*; these findings highlight the pivotal role of DNMT3A in the establishment of T cell exhaustion.^52^ In addition, DNMT3A-KO CAR-T cells demonstrate superior expansion capacity and retain their cytolytic activities after repetitive antigen stimulation. Intriguingly, *in vivo* assessments revealed that the antitumor efficacy of DNMT3A-deficient CAR-T cells is higher than that of their wild-type counterparts. IL-10 was shown to play a role in enhancing the functions of DNMT3a-KO CAR-T cells, as shown by the abolished antitumor effects of double-KO CAR-T cells with DNMT3a deletion.^84^

SUV39H1, which is known as a histone methyltransferase, plays a role in the modulation of chromatin states, affecting gene expression. Specifically, SUV39H1 is responsible for the trimethylation of histone H3 at lysine 9, which is a marker that is typically associated with heterochromatin and transcriptional silencing.^85^^,^^86^ Thus, the activity of SUV39H1 could have implications for the epigenetic reprogramming of CD8+ T cells, influencing their differentiation and function. In chronic infection and cancer, the alteration of histone methylation patterns by SUV39H1 may contribute to the regulation of T cell exhaustion by modulating the accessibility of key genes that are involved in T cell effector function and memory formation.^86^^,^^87^ Understanding the role of SUV39H1 could, therefore, be crucial for developing strategies to reprogram T cells and enhance the efficacy of therapies such as CAR-T cell therapy. A recent study highlighted the benefits of SUV39H1 disruption in CAR-T cell therapies.^88^ The targeted genetic modification of this histone methyltransferase was found to enhance CAR-T cell expansion, persistence, and antitumor efficacy, particularly in leukemia and prostate cancer models. This study highlights the improved performance of SUV39H1-edited CAR-T cells in sustained tumor control, suggesting that epigenetic programming could be key to enhancing the therapeutic resilience and effectiveness of CAR-T cell treatments.^88^ This new research direction indicates that modulating the SUV39H1 pathway could help refine CAR-T cell therapies to improve clinical outcomes.

While the aforementioned transcription factors regulate the differentiation, maintenance, and function of exhausted T cells, persistent antigen stimulation induces epigenetic reprogramming, modulating the expression of essential functional proteins and transcription factors and ultimately leading to the irreversible establishment of epigenetic scars. Consequently, once exhausted, cells maintain decreased cytokine expression and proliferative capabilities even after antigen withdrawal.^89^ Moreover, the stabilized epigenetic landscape of exhausted T cells is distinct from that of memory or effector T cells, illustrating a unique T cell exhaustion fate. This enduring epigenetic scarring inhibits the persistence of T cell rejuvenation or reinvigoration after PD-1 blockade.^9^^,^^90^^,^^91^ Furthermore, the epigenetic scars in exhausted T cells prevent their differentiation into memory cells even after antigen clearance, and the adoptive transfer of sorted memory T cells into tumors induces the reprogramming of the suspended epigenetic state of memory T cells into a dysfunctional tumor-specific state.^92^ Therefore, exploiting epigenetic engineering in CAR-T cell therapies offers a strategic approach to circumvent T cell exhaustion, thereby potentiating the therapeutic potential of these T cells. This approach leverages the plasticity of epigenetic modifications to sustain and enhance the antitumor functions of CAR-T cells, representing a significant step forward in the advancement of cancer immunotherapy.

## Revitalizing exhausted T cells: Innovations in CAR-T cell therapy

**Figure 3:**
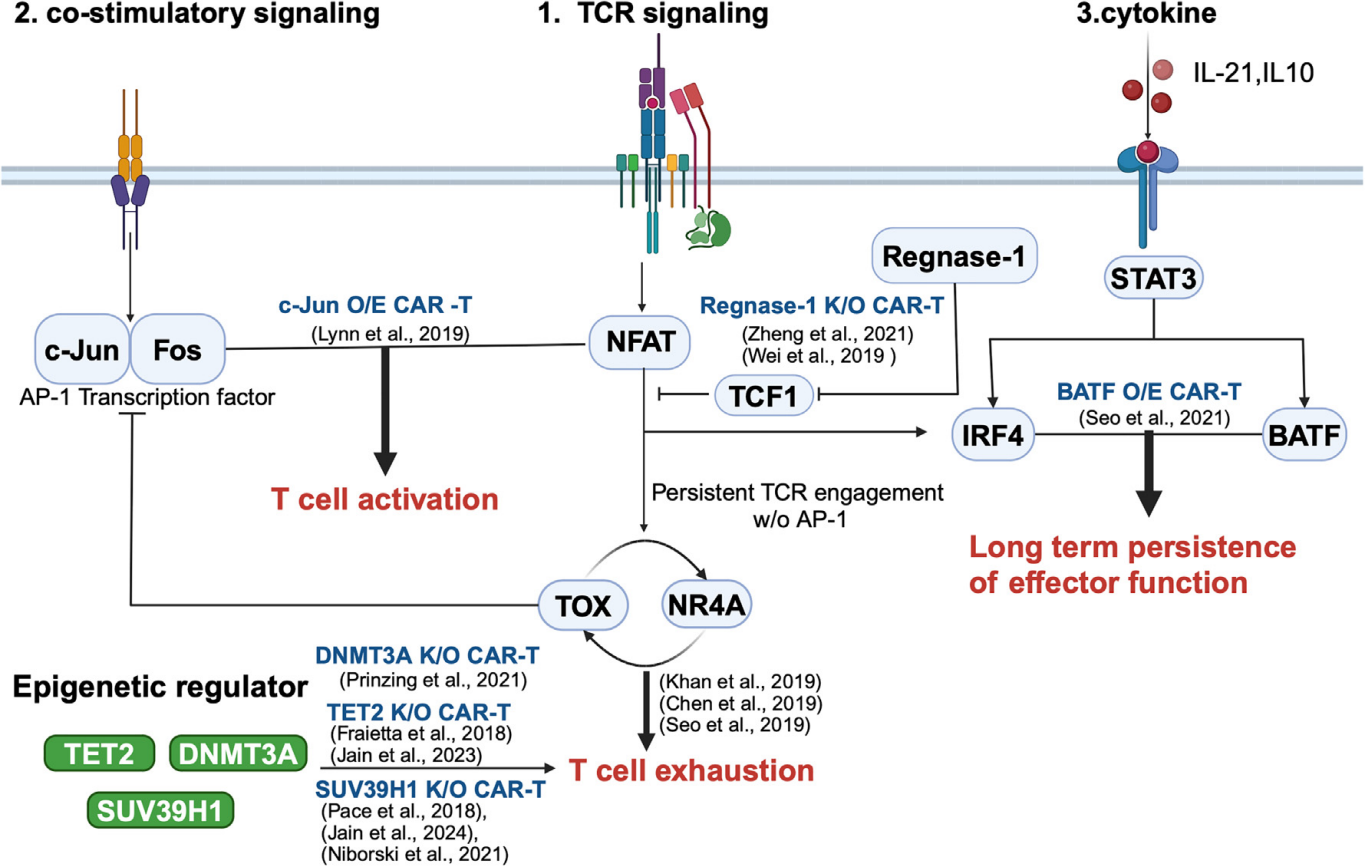
Schematic of external upstream signals and interactions of transcriptional factors and epigenetic modulators This schematic captures the intricate web of upstream signals and their downstream interactions among transcription factors and epigenetic modulators that govern CAR-T cell function. Noteworthy interactions between specific transcription factors that lead to functional consequences are accentuated in red. Blue notations refer to findings from prior research that adjusted these interactions to bolster CAR-T cell efficacy (O/E, overexpression; K/O, knockout). The costimulatory-signal-activated c-Jun forms the AP-1 transcription factor complex with Fos, which, alongside NFAT, triggers TCR signaling and T cell activation. In contrast, without AP-1, NFAT alone can trigger factors like TOX and NR4A, which are pivotal for T cell exhaustion. Concurrently, transcription factors such as BATF, IRF4, and STAT3 are modulated by external cytokine signals and are essential for the effector functionality of CD8+ T cells. Remarkably, BATF and IRF4, following TCR signal reception, collaborate to preserve T cell effector functions. TCF1, abundant in T_ex_^Prog^ cell subsets, dampens TCR signaling, thus aiding in the conservation of the T_ex_^Prog^ cells’ dormant state. Epigenetic regulators like TET2, SUV39H1, and DNMT3A play a significant role in driving T cell exhaustion, presenting potential targets for enhancing CAR-T cell therapy.

Exploring the molecular mechanisms underlying T cell exhaustion and dysfunction has laid a foundation for important research aimed at rejuvenating CAR-T or T cells to increase their therapeutic impacts. Strategies that aim to improve the long-term persistence of CAR-T cells are notably compelling (Figure 3). For instance, knocking out Regnase-1 in CAR-T cells results in an increased proportion of TCF1+ CAR-T cells, which contributes to exceptional long-term persistence and superior antitumor capabilities. These enhancements are attributed to the critical role of TCF1 in the formation, maintenance, and function of the stem-like T_ex_^Prog^ cell subset^93^ (Figure 3).

Furthermore, innovative approaches, such as overexpressing c-Jun, which is part of the AP-1 transcriptional family, have shown promising results in improving the functional and proliferative capacities and *in vivo* antitumor activities of modified T cells^94^ (Figure 3). In parallel, overexpressing the transcription factor BATF has resulted in even more promising therapeutic outcomes, including sustained antitumor responses and increased effector functions.^50^

Considering the interactions among AP-1 transcription factor family members, IRF and STAT, and their reliance on TCR signaling, combinations of these optimal transcription factors may result in enhanced properties in adoptive cell therapy, such as increased effector functions, resistance to exhaustion, and prolonged response capacity.^20^^,^^67^^,^^68^^,^^95^^,^^96^ Given the rapid progression of T cell exhaustion and dysfunction due to antigen persistence and dose-dependent TCR signaling, optimizing respective CARs holds significant promise for improving therapeutic approaches.^97^

CAR-T cells undergo various levels of antigen-independent tonic signaling leading to a state of exhaustion, which is a phenomenon that is observed across cells that express different single-chain variable fragments (scFVs).^27^^,^^98^ Intriguingly, inadequate levels of tonic signaling equally compromise the antitumor efficacy of CAR-T cells.^99^ To optimize such signaling, many innovative strategies are being explored. These strategies include modifications in the optimal costimulatory domain, alterations in the framework region, and adjustments in the complementary determining region of the scFV.^98^^,^^100^^,^^101^ Advances also include innovations such as inducible CAR expression^102^^,^^103^ and the logic-gated selective activation of the signaling pathway downstream of the CD3*ζ* domain.^104^ These groundbreaking strategies aim to increase the therapeutic efficacy of adoptive cell therapy and promote resistance to exhaustion, and extensive and active research in these promising fields is ongoing.
